# Registrars’ experience with research in family medicine training programmes in South Africa

**DOI:** 10.4102/safp.v66i1.5907

**Published:** 2024-04-10

**Authors:** Emcy Louw, Robert J. Mash

**Affiliations:** 1 Division of Family Medicine and Primary Care, Faculty of Medicine and Health Sciences, Stellenbosch University, Cape Town, South Africa

**Keywords:** research, research activities, medical residency, postgraduate training, graduate education

## Abstract

**Background:**

Completion of a research assignment is a requirement for specialist training in South Africa. Difficulty with completion delays graduation and the supply of family physicians. The aim of this study was to explore the experience of registrars with their research in postgraduate family medicine training programmes.

**Methods:**

An explorative descriptive qualitative study. Extreme case purposive sampling selected registrars who had and had not completed their research on time, from all nine training programmes. Saturation was achieved after 12 semi-structured interviews. The framework method was used for data analysis, assisted by ATLAS.ti software.

**Results:**

The assumption of prior learning by teachers and supervisors contributed to a sense of being overwhelmed and stressed. Teaching modules should be more standardised and focussed on the practical tasks and skills, rather than didactic theory. Lengthy provincial and ethics processes, and lack of institutional support, such as scholarly services and financial support, caused delays. The expertise of the supervisor was important, and the registrar–supervisor relationship should be constructive, collaborative and responsive. The individual research experience was dependent on choosing a feasible project and having dedicated time. The balancing of personal, professional and academic responsibilities was challenging.

**Conclusion:**

Training programmes should revise the teaching of research and improve institutional processes. Supervisors need to become more responsive, with adequate expertise. Provincial support is needed for streamlined approval and dedicated research time.

**Contribution:**

The study highlights ways in which teaching, and completion of research can be improved, to increase the supply of family physicians to the country.

## Introduction

Primary health care should be the cornerstone of healthcare systems.^1,2^ In recent years, governments have re-committed themselves to primary health care and the need for primary care research.^2,3,4^ According to the World Health Organization’s 2008 World Health Report on primary healthcare, ‘we need a health system that responds better and faster to a changing world’.^3^ Primary health care research is needed for such a response, to inform evidence-based practice, to improve health systems and policies, and to strengthen service delivery.^4^ Parallel to this, there has been a global decline in physicians involved with research since the 1990s, which in turn, has intensified the focus on research in education programmes.^5^

Primary health care is widely viewed as important, but in many countries it is still poorly developed with a lack of underlying evidence and research.^6^ Primary health care is unique in that it has the potential to improve the health status of populations and enable the health system to be more responsive to community needs, as well as more resilient, efficient and equitable.^7^ A wide variety of research is possible, including basic research, clinical research, research on health services and health systems, as well as education and training of the workforce.^8^

Globally, education in research skills is recognised as a fundamental aspect of residency training for clinicians.^9^ In sub-Saharan Africa, the need to train family physicians has been emphasised, including their ability to perform research.^10^ Family physicians in Africa can make an important contribution to district health systems, and adequate training in all their roles, including research, is vital.^10,11^ Family physicians in South Africa need to fulfil six roles: as care providers, consultants, capacity builders, clinical trainers, clinical governance leaders and champions of community orientated primary care.^12^ All of these roles require insight into evidence-based medicine and appraisal of research for the healthcare team. The research toolkit is also of value in clinical governance to evaluate and improve the quality of care as part of service delivery.^11^ In the Western Cape, family physicians have also created a practice-based research network to conduct relevant applied research projects.^13^

A number of strategies have been identified to help build research capacity in primary health care settings in developing countries.^4^ These strategies include the development of training programmes with a focus on research skills (including mentorship programmes), motivating medical schools to establish family medicine departments that can build research capacity, encouraging primary care clinicians to partake in research activities, and also incorporate research into patient care and service delivery, and to establish partnerships with international organisations that support research.^4^

Echoing this, the Health Professions Council of South Africa (HPCSA) issued a directive in 2011, making a research component a prerequisite for registering as a specialist.^14^ However, this prerequisite now serves as an obstacle to graduating new family physicians and limits the output of the training programmes, as many programmes lack supervisory capacity and established researchers. A recent analysis of family physicians in South Africa concluded that a three-fold increase in throughput is needed to fulfil the minimal needs of the health system for family physicians.^15^ Improving the completion of the research assignment will help to achieve this goal.

Literature on the experience of registrars with conducting research is scarce, and most evidence is from high-income countries. Factors identified as barriers to research in the primary care setting include: time constraints, lack of research skills, lack of research training or curriculum, lack of adequate supervision, and balancing clinical duties with research.^14,16,17,18,19^ There is evidence that training is needed not only in undertaking research, but also in understanding and using research in daily practice.^19^ Key factors that influence the success of registrars in performing research include: availability of mentorship, adequately qualified supervisors, training programmes that focus on orientation and preparation for research, developing skills in evidence-based practice (even at an undergraduate level), opportunities to publish and present research, partnerships with health services and policy makers to facilitate support for research, adequate scientific software and financial resources.^16,20,21^

In South Africa, a recent study with surgical registrars identified a few factors that impede the research process.^22^ These included a lack of funding to perform research, a lack of dedicated time to complete the research and the burden of clinical responsibilities. No such study has been performed with family medicine registrars and key questions remain – Is there adequate teaching, support and supervision? How does undergraduate training influence capability as a registrar? What factors influence the registrars’ success in their research assignment? How can programmes improve the registrars’ capacity to complete research assignments on time? The aim of this research was to explore the experience of registrars with their research assignments in postgraduate family medicine training programmes across South Africa. Specific objectives were to explore the registrars’ prior learning of research skills, their experience of formal teaching of research, the challenges of balancing clinical responsibilities with doing research, the registrar-supervisor relationship, and the influence of the institutional environment on performing research.

## Methods

### Study design

The study was designed as an explorative descriptive qualitative study sectional, qualitative study in order to understand the experience of registrars with their research assignments.

### Setting

Registrars in family medicine are trained through nine universities in South Africa: the University of Cape Town, the University of Witwatersrand, Stellenbosch University, the University of KwaZulu-Natal, the University of Pretoria, Sefako Makgatho University, the University of the Free State, the University of Limpopo and Walter Sisulu University. Each university has a number of accredited training complexes in both rural and urban settings. Registrars are mostly trained in primary health care and district hospitals, but can also rotate to larger regional and tertiary hospitals. Training programmes are for 4 years and registrars attempt to take the Fellowship of the College of Family Physicians (FCFP) exit examination in their fourth year. At the time of this study, Part A of the Fellowship was a clinical examination and Part B required successful completion of the research assignment. If a registrar had not completed their research assignment, they could not obtain the Fellowship or register as a family physician with the HPCSA. Completion of the research assignment is therefore a rate-limiting step in the national pipeline of new family physicians. A recent decision by the College of Family Physicians will remove the research requirement from the Fellowship examination sometime in the future, but this will remain a requirement for the HPCSA.

Each training programme has its own approach to teaching research and supporting novice researchers. Universities may start the process at different stages of the programme and have varying levels of supervisory capacity. Universities may also differ in the way research is approved and ethical issues are considered. They may also differ in the specific requirements of the final research assignment, as some may require a journal article format and others a formal dissertation. The experience of registrars and success in completing their research may, therefore, vary considerably between programmes.

The researcher was a final year registrar in family medicine and was working in a rural town as part of the training programme at Stellenbosch University. The researcher acted as interviewer, and interviews took place on a virtual platform. The researcher had not performed qualitative research previously and was supervised. In performing this research study, she had to experience many of the same challenges that were described by the participants and realised that this was a difficult experience for many of her colleagues. She had no prior experience of registrars from other training programmes. She did not know registrars from the other university programmes. She was aware of the need to remain open to all perspectives in the interviews and analysis, and to be aware of her own viewpoints.

### Study population

The study population included newly graduated family physicians (having passed FCFP exams within the past year) and registrars still in their final year (year 4) of study. Approximately 20 new family physicians pass the exam each year, and there are around 55 registrars nationally per year in the programmes.^15^ There were no exclusion criteria.

### Sample size

The intended sample size was 18 registrars, with 2 registrars from each university. Concurrent data analysis determined the final sample size. Initial analysis was conducted after the first nine interviews (one per programme), and if thematic saturation was not achieved, then five more interviews were performed, again followed by data analysis. The final four interviews were only performed if saturation was still not achieved, with the possibility of additional interviews if necessary.

### Sampling strategy

Extreme case purposeful sampling was used to identify one registrar who completed research in a timely way (defined as completing research in time to take the Fellowship Part B in year 4 of the programme) and one registrar who needed extension to complete research (defined as not completing in time to pass Part B of the Fellowship during their fourth year of the programme).

Participants were selected with the help of their representative on the South African Academy of Family Physician’s (SAAFP) Education and Training Committee. Registrars have formed a virtual community in South Africa, and their representative on the SAAFP is in touch with registrars from all the programmes. The representative on the SAAFP was contacted to help identify suitable candidates for the study; an informative message was sent to the proposed candidates via the representative, only once the candidate agreed to partake in the study was their information shared with the interviewer.

### Data collection

The researcher developed an interview guide with potential open-ended questions on each topic (Table 1). These topics covered all five objectives of the study and were informed by the available literature.^5,9,21^ The guide was revised with feedback from the supervisor, was piloted, reviewed and subsequently approved.

**TABLE 1:** Overview of the interview guide.

Topics	Issues to cover
**Topic 1: Undergraduate training**	
Q: Describe your undergraduate training in research and any prior involvement in research.	Undergraduate training Involvement in previous research Additional training
**Topic 2: Formal teaching**	
Q: Describe your experience with formal research teaching in your MMed programme	Dedicated research module Dedicated time to complete proposal and research assignment Guidance from tutors
**Topic 3: Clinical responsibilities**	
Q: What was your experience in balancing research work with clinical responsibilities? What were hindrances and what was helpful and why?	Time constraints Attitudes of co-workers to research Assistance from colleagues with research assignment Dedicated time for research Co-supervisors if any
**Topic 4: Registrar-supervisor relationship**	
Q: How would you describe the registrar-supervisor relationship you experienced?	Who identified the supervisor – appointed or chosen Background and expertise of the supervisor Accessibility and frequency of contact Mode of communication and interaction Usefulness of feedback and guidance How were any problems in the relationship handled
**Topic 5: Institutional involvement**	
Q: What was your experience of the institutional processes regarding your research?	Ethics process and approval Administrative support Library services Scholarly support services Statistical support Funding support/grants Examination requirements and process

The researcher contacted participants in advance and sent a consent form together with an information sheet pertaining to the research. Semi-structured interviews took place at a mutually agreeable time, in English, via a virtual platform (Zoom), over 30–60 min. All interviews were audio-recorded for later transcription and analysis.

### Data analysis

A professional transcriber created verbatim transcripts, and the researcher checked them for accuracy against the audio tapes. The researcher performed the analysis with supervision of key steps. Concurrent analysis followed the steps of the framework method with the assistance of ATLAS.ti software.^23^ The framework method was developed for applied policy research and has been used extensively by family medicine in qualitative studies within South Africa. It provides a clear and structured approach to qualitative data analysis:

1. Familiarisation: the researcher reviewed the recorded interviews and transcriptions, and inductively identified issues that could be coded.
2. Coding index: codes were defined from the inductive issues that were identified from the data in step 1. They were then organised into categories.
3. Coding: codes were applied to each transcript.
4. Charting: codes were grouped into families within ATLAS.ti according to the categories developed in step 2. Reports were created in ATLAS.ti that brought all the data together within a code family.
5. Interpretation: each report was interpreted to derive themes and subthemes. The range of opinions and experiences in each theme were described as well as any relationships between themes.

The supervisor assisted with checking the coding index and the interpretation as well as assisting with using ATLAS.ti software to perform the analysis. Reflexivity formed part of this process, and the author’s own reactions and thoughts to the data were documented and discussed with the supervisor.

### Ethical considerations

Ethics approval was obtained from the Health Research Ethics Committee 1 of Stellenbosch University (reference number S21/07/114). The study was conducted according to the ethical guidelines and principles of the International Declaration of Helsinki, South African Guidelines for Good Clinical Practise and the Medical Research Council Ethical Guidelines for Research. The national Education and Training Committee of the SAAFP gave permission to conduct the research.

## Findings

Table 2 describes the characteristics of the participants. The different training programmes are identified by a letter, to show the distribution across programmes, but not the actual programmes. Thematic analysis identified seven themes with subthemes as shown in Table 3.

**TABLE 2:** Characteristics of the participants.

Interview	Age (years)	Gender	Position at time of interview	Completion of the research	Training facility
Interview 1	37	Male	Newly qualified family physician	Completed during registrar time	A
Interview 2	36	Male	Registrar year 4	Completed during registrar time	B
Interview 3	31	Female	Registrar time completed	Still completing	C
Interview 4	32	Male	Newly qualified family physician	Completed during registrar time	D
Interview 5	34	Male	Registrar time completed	Still completing	B
Interview 6	36	Male	Newly qualified family physician	Completed during registrar time	E
Interview 7	43	Male	Registrar time completed	Still completing	F
Interview 8	32	Female	Newly qualified family physician	Completed during registrar time	G
Interview 9	32	Male	Newly qualified family physician	Completed after registrar time	A
Interview 10	36	Male	Newly qualified family physician	Completed during registrar time	F
Interview 11	39	Female	Registrar time completed	Still completing	H
Interview 12	36	Female	Registrar year 4	Still completing	I

**TABLE 3:** Themes and subthemes.

Themes	Subthemes
Prior exposure to research	Minimal prior exposure to research Assumption of prior knowledge and competencies led to feeling overwhelmed and emotional distress
Formal teaching of research competencies	Formal programmes were offered by training institutions, but with no standardised approach Additional training courses led to an improved skillset Journal clubs and contact sessions improved research competencies
The institutional processes involved with the research process	Ethics and provincial approval was a time consuming process Library services and scholarly support services were crucial for the completion of the research project Lack of transcription and translation services led to outsourcing Lack of financial support led to self-funded research and subsequent delays The aim to publish was a positive driving force to complete the research Fair examination process
The experience of performing research	The feasibility of the research project was a deciding factor in the completion thereof Lack of deadlines Challenges of the health system Supportive resources such as research assistants Informative process with the learning of new skills and research competencies
External and unpredictable factors influencing the research process	Coronavirus disease 2019 (COVID-19) pandemic offered challenges and opportunities Social unrest and riots caused challenges and delays
The registrar-supervisor relationship	A collaborative approach was needed with the appointment of the supervisor Responsiveness, accessibility and quality of feedback was vital Supervisor could also function as a mentor and role model Expertise of the supervisor contributed to the trustworthiness of the relationship
Balancing clinical responsibilities with personal factors and the research process	The challenges of balancing clinical responsibilities with the academic load and personal factors Time taken away from clinical duties could cause strained workplace relationships Protected time to perform research supported the timely completion of the project Registrars had to navigate stress, the emotional burden, time management and motivation related to the research project Experience of performing the research project influenced the possibility of future engagement with research

### Prior exposure to research

There was little undergraduate exposure to performing research. Most of the registrars had never been involved with research or exposed to the research process. Unlike the registrars’ clinical skills, it was important that teachers did not assume prior learning of research methods or processes. Registrars were mostly complete novices in the field of research:

‘So I would say for someone who knows as little as we do at our first or second year of our Masters training, I would say, [I] mean I think people forget that we really do not know anything about research.’ (37-year-old male, newly qualified family physician, completed the research on time)

Participants reported that this limited experience with research contributed to feeling overwhelmed by the requirements of the research assignment. Few participants reported being involved with aspects of research prior to starting the programme. For example, taking part in clinical audits, writing articles or helping to collect data for someone else’s study, but none of these experiences fully prepared them for the research assignment. However, those few participants who were involved with prior research, reported an improved experience with performing the research assignment.

### Formal teaching and training of research competencies

A formal teaching module on research typically took place in the first or second year of training. One participant reported that there was no formal teaching and that all teaching was informal by family physicians at the training sites. Formal modules were quite different in content, and might focus on teaching research methods, critical appraisal skills, or writing the research proposal. The educational approaches also differed, from didactic teaching to round table discussions or peer review groups. The feedback from respondents suggested that feedback on their draft proposals and research assignments as well as interactive discussions were of more value than just learning theory about research:

‘We spend the first year having qualitative meetings where [we] would present a proposal or something like that, and people would ask questions. Fine-tuning so that by the time you get going you got quite a mature idea of what you were wanting. Rather than running ahead with something that you hadn’t quite whittled down to what could be achievable.’ (36-year-old male, newly qualified family physician, completed the research on time)

Outside of the departments of family medicine, universities and other organisations offered optional additional training courses on research skills. Many participants did not consider such additional training, but registrars who did, reported an improved skill set. Topics ranged from performing and analysing qualitative research to courses focussed on biostatistics. Complementary to formal teaching of research, the participants also noted that journal clubs and contact sessions contributed to their overall learning. Journal clubs were valuable as they exposed registrars to research studies, helped them appraise and interpret research, and then applied their new knowledge to their own research assignments. Although journal clubs or contact sessions were used by participants to help them complete their proposals and data collection, they could not replace formal teaching of research.

### The institutional processes involved with the research process

Participants experienced the process of obtaining ethics approval as a time-consuming barrier to completing the research assignment, which caused significant frustration. On the other hand, many participants were aware of this barrier, and most were not surprised at the time between submission to ethics and receiving feedback. Likewise, many registrars required provincial permission to perform their research within the health services, and all agreed that this too was a time-consuming process:

‘I had a big delay when I submitted my proposal to ethics, I think had about a four- or five-months delay to get it back. And so that was frustrating for me.’ (36-year-old male, newly qualified family physician, completed the research on time)

Library services played an important role in completion of the research proposal. Participants often consulted dedicated library personnel to assist with searching for and finding articles relevant to their research subject. Feelings of gratitude and relief were common. The use of scholarly support services included biostatistics, transcription, copyediting and translation services. Participants reported that the value of statistical support was dependent on the statistician involved. Transcription and translation services were not readily available and on occasion had to be outsourced. The experience of copyediting services was overall positive and contributed to a more professional presentation of the research assignment.

The financial burden of performing a research assignment was well managed by the registrars. Typical costs included ethics review, travel, cell phone data and transcription services. Some participants were awarded funding through their universities or from other organisations, but most studies were self-funded. In the case of self-funded studies, the participants managed to complete the studies without financial support even though this did on occasion cause a delay in the process:

‘I had to look in private if I can get such a person. And they don’t come cheap. Yes. And the research is self-funded, so that was the other delay.’ (36-year-old female, registrar year 4, still completing the research)

The possibility of publishing the research added value to the investment of time and energy in the research assignment. Some registrars were motivated by the possibility of publishing their work and opportunities to engage with colleagues who shared the same interests at conferences and academic days. The format of the final assignment differed across training programmes from full dissertation to publication-ready articles. It was evident that publication-ready formats added extra motivation to complete the research. Although not all participants had experienced the examination process, those who did reported it being a fair process with positive feedback.

### The experience of performing the research

The feasibility of the research project was a common theme. While the research assignment is needed to fulfil the requirements for the Master’s degree, it should also be an appropriately sized task. Various factors enabled participants to choose an achievable goal, such as supervisor feedback and peer review in formal feedback sessions. This feedback enabled the registrars to downscale if needed and simplify their projects to ensure a more feasible study was attempted:

‘And then another factor that I think was really important is that it is your MMed and not your PhD. You need to finish it and it needs to be achievable. It doesn’t [need] to be life altering research, but it does need to be a good experience in terms of getting some research out. And even if it is something that you can publish. It needs to be a good first experience. To make sure that you are able to put limits on what you are researching.’ (36-year-old male, newly qualified family physician, completed on time)

A lack of specific deadlines could contribute to procrastination and delay in the completion of the research assignment. On the other hand, one participant reported that the pressure of a deadline to choose a topic caused him to choose a topic that he was not particularly passionate about, leading to poor motivation to complete the project.

Data collection in the health system could be challenging. For example, the incomplete nature of clinical note keeping and disorganisation of the filing system complicated accurate and complete data collection, and delayed the process.

Registrars also reported specialist departments agreeing to assist with participant identification and data collection, but then not following through. One registrar even reported that they were required to take up additional managerial responsibilities to act as the interim clinical manager in a rural hospital, which delayed the completion of the research assignment.

A research assistant was mentioned as a helpful resource. Finding a suitable assistant could be difficult, especially when there were language requirements. The use of an assistant could be a methodological requirement in order to reduce bias or overcome language barriers and could also be necessary to make progress while still fulfilling one’s clinical responsibilities. However, research assistants also needed to be paid, trained and supervised:

‘…it can be quite hard to actually get a research assistant, for example, if you do need to acquire the data during normal working hours, and don’t want to impact your service delivery, etcetera. To try and actually get a reliable research assistant and to get funding for such a person can be quite a challenge.’ (32-year-old male, newly qualified family physician, completed the research on time)

Nevertheless, respondents all agreed that the experience of performing their own research was an informative and learning experience with many new skills and competencies acquired through the process.

### External and unpredictable factors influencing the research process

According to respondents, the COVID-19 pandemic influenced the completion of their research projects in various ways. For some participants, the pandemic created new hurdles; for example, some participants had to conceptualise new research projects when their original projects were no longer feasible with COVID-19 restrictions:

‘But, you know, those are the sorts of unforeseen things that happened with research, predicting global pandemics isn’t exactly easy when designing your questionnaires etcetera.’ (32-year-old male, newly qualified family physician, completed the research on time)

The COVID-19 pandemic also saw respondents taking up new roles such as sub-district COVID-19 coordinators, increasing their clinical responsibilities and limiting time for the research even further. For others, the pandemic created new opportunities for research. For example, studies involving the homeless population, who were now housed in shelters during hard lockdown, and were easily accessible as a study population. Furthermore, unforeseen incidents such as riots and social unrest hampered data collection for other participants.

### The registrar–supervisor relationship

Programmes differed in how they appointed supervisors. Some registrars mentioned being assigned a supervisor with no input from their side and found this to be an authoritarian approach by the respective department or faculty. On occasion, the appointment was made based on ease of access, as the supervising family physician worked at the same facility. On some occasions, it was a collaborative decision, based on the interests and expertise of the available supervisors, in conjunction with the topics presented by the registrars. The registrar’s personal choice was also considered in some instances. Ease of access and responsiveness was highly valued by the registrars:

‘Supervisors are your family physicians who are quite readily available and who you work with quite often so you can always contact with them as well.’ (43-year-old male, completed registrar time, still completing the research)

Furthermore, if the registrar’s personal choice and expertise of the supervisor were considered, it led to greater confidence in the supervisor and improved the overall experience with the research process.

Some of the common complaints mentioned by the participants related to the accessibility and responsiveness of the supervisors. Most reported that supervisors were easily accessible, via email or telephone, sometimes in person, but that the responsiveness to aspects of the research was inconsistent. Some supervisors were very responsive towards questions, while others took longer than the agreed amount of time (varying in weeks up to months) to respond to queries, and others did not respond at all:

‘So I felt so alone because you write something, send Prof, he’ll take like months to respond to you, and remember your programme is not research alone.’ (39-year-old female, completed registrar time, still completing the research)

Several potential factors were mentioned as contributing to the responsiveness of the supervisor. Other research responsibilities, for example, being supervisor to many students, could limit responsiveness. Other work responsibilities, such as clinical responsibilities in the case of a family physician or managerial responsibilities as head of department, might also impede supervision:

‘I guess we need to understand that also, they are not just there for research and my supervisor in particular, I think he had six or seven research people that he was helping.’ (43-year-old male, completed registrar time, still completing the research)

Some participants reported that the feedback from supervisors was not constructive and did not contribute to the value of the final assignment or help them learn new skills:

‘The person would not try and help with small details or help to try and find where I’m struggling to see if they can help with that.’ (37-year-old male, newly qualified family physician, completed the research on time)

Other respondents experienced the complete opposite in terms of communication with their supervisor and the support offered to them during this assignment. They reported having support throughout the process; for example, the supervisor being always available and giving continuous feedback as the project developed. This improved the registrar’s ability to continue with the project in a timely way. Such supervisors gave special attention to guiding the registrar through the research process, for example, investing time and energy to ensure the development of new skills and competencies:

‘…she actually links me to also some of her colleagues who [are] experienced in certain things that if she’s not sure about you know, she’ll refer me to them and then we will engage with them in our conversations in how to approach the research.’ (31-year-old female, registrar time completed, still completing the research)

In cases where the supervisor gave constructive feedback and offered ongoing guidance, the respondents felt more supported and had an improved experience. Constructive feedback also enabled the respondents to learn and practice new skills, such as adopting an academic writing style early in the write up of the assignment.

The registrar–supervisor relationship was described as a collaborative effort and an interdependent relationship, where the participant works on an element of the project, sends it to the supervisor and then needs to wait for feedback before being able to continue with the work on the assignment. Sometimes this iterative process could be too slow and disrupt momentum:

‘Then making changes, sending it back, then, you know, waiting another two, three weeks just to get minor changes back. So that, yes that’s why I said, it felt quite tedious at a stage, trying to make changes but only getting a reply on your changes a month later. And then you’re already kind of forgot and your focus has already shifted to something else.’ (32-year-old female, newly qualified family physician, completed the research on time)

The expertise of a supervisor was another contributing factor to the overall experience of participants and the quality of the research assignment. Supervisors were perceived as more effective if they had prior experience of supervising students, and experience in performing quantitative or qualitative research:

‘And I can say that the quality of your research also depends on your supervisor’s experience. So the more experienced supervisor you have, the better experience you have in terms of doing the research because they are able to guide you better.’ (31-year-old female, registrar time completed, still completing the research)

Some respondents alluded to the supervisor having, not only a supervisory and capacity-building role in the research assignment, but also developing a broader mentoring role for the registrar. Respondents reported that such supervisors could be a role model and mentor for the family medicine specialty or fulfilling the role of a ‘father figure’. Other participants reported that the supervisor did not fulfil the role of mentor and that a more in-depth relationship was needed to develop a mentor–mentee relationship. The exit of a supervisor, for example, by resigning or taking up another post, complicated the research process. This supervisor might become less accessible and responsive or a new supervisor might be needed.

### Balancing clinical responsibilities with personal factors and the research process

Respondents reported that the family medicine training programme had many academic tasks and responsibilities. For example, assignments, presentations, quality improvement projects (QIP), self-studying and portfolio work (including observed consultations and observed procedures):

‘So, family medicine has a lot of coursework, a lot of assignments, a lot of mini research projects. I mean, the QIP itself is a research project. So, there’s a lot of content in terms of how much you have to learn, and how much you have to do. And then on top of that, the research is also quite demanding, it’s an additional thing.’ (31-year-old female, registrar time completed, still completing the research)

This research project was often happening simultaneously and then neglected in favour of the immediate academic tasks. Registrars found it challenging to balance all their responsibilities related to the specialisation, because of the multifaceted nature of the programme as well as other responsibilities such as clinical work, overtime hours, and personal and family life. The urgent nature of clinical work led to it often taking preference over performing the research component:

‘I think the crux of the matter is that the research often feels like it takes a back foot to the rest of your, your responsibilities as a registrar.’ (32-year-old male, newly qualified family physician, completed the research on time)

The reality of the health system is that registrars take up a clinical post, and therefore, if they take time out for research, clinical service delivery is directly influenced, and the clinical team will have to carry the consequences. This led to conflict between the clinical and academic responsibilities. The responsibility of performing research influences the registrar’s relationship with co-workers. Registrars often felt guilty for not pulling their weight in the clinical area when they had to dedicate time to performing research and were unable to fulfil their clinical duties. This contributed to strained relationships in the workplace:

‘So it does sort of create a [strained] relationship between registrars and other colleagues because people feel that you actually have time off when you actually don’t have time off, you actually have so much on your plate and people just feel like you’re just dodging and diving the whole time and you are never there, you know so it does create a very questionable relationship with colleagues.’ (31-year-old female, registrar time completed, still completing the research)

However, when co-workers were actively involved in the research, such as assisting with identifying participants, the experience could change from strained to a supportive and collaborative relationship.

Participants had varied experiences in obtaining protected time to perform their research. Some respondents reported that protected time was a theoretical concept and never realised in practice. Whereas other respondents used dedicated time (designated for different aspects of the programme such as journal clubs, contact session and special leave) to facilitate various components of the research process in order to not have it compete with clinical responsibilities. The shared expectation among participants was that dedicated time should be a priority and was a necessity for successful and timely completion of the research assignment:

‘I feel like something that must be set out from the very beginning for registrars is, “if you need to complete an academic program for which your research it is such a big component of; the least you can get is time to do it during the day.” Because it is very difficult to complete something like this. It is such a major part of completing your degree.’ (32-year-old female, newly qualified family physician, completed research on time)

Much of the research was completed at home or after hours, where it competed with commuted overtime as well as family and personal life. Personal factors influencing the research assignment included issues such as pregnancy and childcare, maternity leave as well as health issues. Registrars needed to cope with stress, stay motivated, handle the emotional burden of performing the research and hone their time management skills. It was easy to become overwhelmed, and this could also impact on completion of the registrar programme as a whole:

‘So it gets overwhelming, extremely overwhelming. It gets extremely tiring. And I think that’s one of the reasons why we lose so many registrars in the course people just don’t finish the course because it’s, it just gets overwhelming and people just don’t cope with the demand of, of the course you know, so, yes, so it was, it’s not easy. It’s not easy at all.’ (31-year-old female, registrar time completed, still completing the research)

Completion of the research assignment depended greatly upon the motivation of the individual registrar. This motivation stemmed from self-driven work and learning. Participants agreed that motivation played a large part in getting the work done. Choosing a topic that maintained and inspired such motivation was a key factor. A respondent also reported that this motivation was linked to the registrar–supervisor relationship and that support and feedback from the supervisor could influence the motivation with which the registrar approached the research project.

Participating in the research assignment had the participants divided in terms of their future likelihood of performing research. Some respondents would grasp at the opportunity to go through the process of doing research again. Other participants shared that this experience has demotivated them to perform research in the future, but given the opportunity to improve the circumstances surrounding the research process they would consider engaging with research again.

## Discussion

### Summary of key findings

**FIGURE 1:**
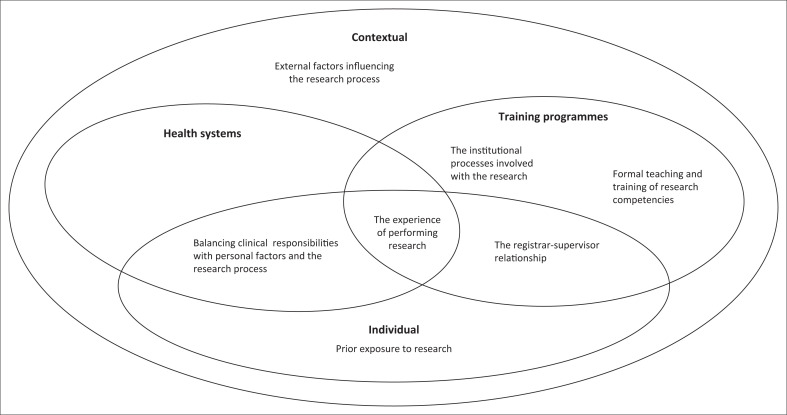
The interconnected nature of the factors influencing the research experience.

The successful and timely completion of the research assignment for the Master’s component of the Family Medicine training programme in South Africa is a complex and interdependent process (Figure 1). Multiple factors are important and interact: the registrar’s prior exposure to research, the teaching of research skills, the academic institutional processes, the individual experience of performing the research assignment, and the balancing of clinical duties with academic and personal responsibilities. Furthermore, the registrar–supervisor relationship was a critical component. External unpredictable factors, such as the COVID-19 pandemic and social unrest, could also impact on the process. As shown in Figure 1, the interconnectedness of these factors is evident. The training context and health system, where the registrar worked, also contributed to the overall research experience. Universities that focussed on offering formal teaching appropriate to registrars, a collaboratively selected registrar–supervisor relationship with adequate institutional support facilitated an improved research experience. These multiple factors and the complexity of the process have been recognised in other international studies also.^16,20,21^

### Discussion of key findings

Registrars are usually building on prior learning when they develop their clinical skills, but this was not the case with the research assignment. Although registrars are enrolled for a Master’s degree, they have minimal research experience as an undergraduate, and are less prepared than other Masters-level students.^24,25^ Supervisors appeared to have an incorrect assumption that registrars had prior learning, whereas most were coming to this as a completely new and daunting task. This led to feelings of inadequacy, incompetence and of being overwhelmed. For many, this research assignment was their first interaction with the research process and could influence their future professional identity as a researcher.

Family physicians have a role to fulfil as researchers in the district healthcare system and being competent in performing research contributes to excellence in their roles as mentors, teachers, clinical governance leaders and capacity builders.^10,11,12^ The development of research competencies is essential to success,^17^ and should be intentional, structured and incremental. Teaching programmes need to focus on building research competencies.^4,9^ Limited research and supervisory capability have been identified as a key factor in several specialist training programmes.^24,25^ There is a need to build such capacity through training opportunities,^24^ collaborative cohort models of supervision,^25^ further training at a doctoral level,^26^ practice-based research networks^13^ and mentoring.^27^

Although participants reported formal teaching of research competencies, there was no standardised format across training programmes. A lack of a uniform approach and agreement on what is required is a widespread problem.^25^ It appeared that a focus on the practical steps of completing the research was more useful than a didactic, theory-based approach to research that was unrelated to the immediate task. Teaching should recognise the lack of prior learning, focus on methods, be appropriate to the disciplinary field, and tailored to the learning needs.^25^ Completion of a submission-ready manuscript as the final product shortens the time taken to complete research by eight months and enables publication.^28^ Modular approaches, forums to present projects and blended learning with digital technology may be useful educational strategies.^25,29^

The institutional processes often led to delays in the completion of the research assignment. Various components were identified: a time-consuming ethics application process, an equally lengthy process to apply for provincial approval, lack of availability of transcription and translation services, and a lack of financial assistance. Even though participants managed to navigate the lack of financial assistance, this has been recognised as a significant barrier.^22^ Registrars have a need for technical assistance in navigating the research journey that may, for example, include administrative or statistical support.^29^

The process of appointing the supervisor, the accessibility and responsiveness of the supervisor, the quality of feedback, and their competing responsibilities, all contributed to completion of the research assignment. In the case of a supervisor with adequate expertise, effective communication, constructive feedback and role modelling, the respondents reported an improved research experience. The importance of these characteristics of effective supervisors has been noted elsewhere.^30,31^

External and unpredictable factors also contributed to various delays in the research process. The COVID-19 pandemic impacted many of the respondents as many research projects were put on hold and teaching was also disrupted.^32^ In the South African context, it is likely that future challenges may involve community protest and unrest, or climate-related challenges.^33,34,35^

Registrars occupy a clinical role with clinical responsibilities, and the balancing act between clinical duties, a heavy academic workload, commuted overtime, family responsibilities and performing research was seen as very challenging. The burden of clinical responsibilities and a lack of time are identified as the commonest obstacles to completing the research assignment.^22,36^ The health services need to acknowledge the time required for the research component and allow dedicated blocks of time and leave for this purpose.^24,25^

Delay in completing the research assignment is one of the key factors reducing the throughput of registrars and the supply of new family physicians. Improving the research process, therefore, will contribute to meeting the goals set by the SAAFP for an increased supply of family physicians at district hospitals and health centres.^37^ Improving the quality of research will also avoid the trap of producing a stream of low quality unreliable or invalid evidence that may do more harm than good when published or presented to policymakers.^25^

### Strengths and limitations

Extreme case purposive sampling enabled a balance of respondents between those who did and did not complete their research on time. Saturation of data was achieved before all the planned interviews were conducted. The researcher conducted the interviews herself, was familiar with the content, and judged that no new themes were emerging in the last three interviews. Although all nine training programmes were represented in the data, three programmes had two respondents, and six had only one respondent. The three universities with multiple respondents had the largest output of new graduates (personal communication from SAAFP) and registrars with both completed and uncompleted research were interviewed. It is likely, therefore, that the findings are a valid exploration of the research experience. Overall, seven respondents had completed their research and five had not. Two of the final interviews were with those that had not completed and contributed to the decision on saturation of data.

The researcher, who also acted as an interviewer for data collection, is a registrar in family medicine and was going through the process of performing her own research during the data collection phase. Although extra care was taken to remain neutral and to prevent her own opinions and experiences to influence the data, the researcher is aware of possibly influencing the interpretation of the views and insights shared during the interview process. Two of the respondents were also known to the researcher and could influence the interpretation of the data. Reflecting on the researcher acting as interviewer, the advantages included an improved understanding of the context of the interviewee. The second author (R.J.M.) supervised the interview and analysis processes, which ensured a high level of reflexivity throughout and ameliorated any loss of objectivity.

Because the research was performed across all training programmes in South Africa, the findings should be transferable to these programmes. Although the data is only from the South African context, the findings could be transferred to training programmes in similar contexts in other African, low- or middle-income countries.

### Implications

The following recommendations are evident from the findings:

- Teaching should focus on assisting registrars with the incremental and practical steps involved in the research journey and provide sufficient theory to support completion of these tasks. As opposed to generic and didactic teaching about research methodology that is unconnected to the registrar’s actual study design and learning needs at that moment. Teachers should be aware of the lack of prior learning in this area and the need to deconstruct the research journey so that it is less overwhelming.
- The registrar–supervisor relationship is critical. The supervisor must have sufficient research expertise and be responsive to communications. Registrars would also value shared decision making in the appointment of supervisors. Institutions should provide ongoing faculty development in postgraduate supervision.
- Institutions need to improve the timeliness of ethics review as registrars have a limited timeframe and their studies are usually small scale and of low ethical risk.
- Provinces should ensure that registrar research is supported, by giving permissions in a timely fashion and enabling opportunities for dedicated time (e.g. special leave) for key aspects of the research. Managers and supervisors should be more aware of the difficulties in balancing personal, professional, and academic responsibilities.
- Registrars would have appreciated more opportunities for financial assistance with research costs from the universities.

Expanding the research question, to include the views of the supervisors, would be of value to explore this phenomenon in further depth. Further research could quantify the issues raised here, and this could provide additional evidence for training programmes. This study did not specifically explore differences between rural and urban training complexes, and this could be a focus of future research. It would also be of interest to replicate this work in other specialist training programmes and determine if these issues apply more broadly.

## Conclusions

The successful and timely completion of the research assignment is a complex problem. Lack of prior exposure to research made the assignment feel overwhelming, and this needs to be addressed in both teaching and supervision. The supervisor–registrar relationship was central to success and supervisor’s needed expertise and responsiveness. Registrars would like more shared decision-making in the appointment of supervisors. Formal teaching should be tailored to the practical steps and tasks of the research journey, and not just provide generic didactic teaching on research methodology. Institutions need to be supportive, through efficient processes for ethics, permissions and opportunities for small scale funding. Strategies are needed to cope with the competing demands of clinical work, personal life, academic tasks and research – dedicated time and special leave can assist with this. Training programmes should take note of the issues raised by registrars and consider revisions to their teaching and management of the research journey.
